# A high-sugar diet rapidly enhances susceptibility to colitis via depletion of luminal short-chain fatty acids in mice

**DOI:** 10.1038/s41598-019-48749-2

**Published:** 2019-08-23

**Authors:** Michael Laffin, Robert Fedorak, Aiden Zalasky, Heekuk Park, Amanpreet Gill, Ambika Agrawal, Ammar Keshteli, Naomi Hotte, Karen L. Madsen

**Affiliations:** 1 Department of Surgery, University of Alberta, Edmonton, Alberta, T6G 2E1 Canada; 2 Department of Medicine, University of Alberta, Edmonton, Alberta, T6G 2E1 Canada; 3 The Centre of Excellence for Gastrointestinal Inflammation and Immunity Research (CEGIIR), University of Alberta, Edmonton, Alberta, T6G 2E1 Canada

**Keywords:** Acute inflammation, Microbiome, Inflammatory bowel disease

## Abstract

Western-style diets have been implicated in triggering inflammatory bowel disease activity. The aim of this study was to identify the effect of a short-term diet high in sugar on susceptibility to colitis. Adult wild-type mice were placed on chow or a high sugar diet (50% sucrose) ± acetate. After two days of diet, mice were treated with dextran sodium sulfate (DSS) to induce colitis. Disease severity was assessed daily. Colonic tissues were analyzed for cytokine expression using the MesoScale discovery platform. Intestinal dextran permeability and serum lipopolysaccharide levels (LPS) were measured. Gut microbiota were analyzed by 16s rRNA sequencing and short chain fatty acid (SCFA) concentrations by gas chromatography. Bone marrow-derived macrophages (BMDM) were incubated with LPS and cytokine secretion measured. Mice on a high sugar diet had increased gut permeability, decreased microbial diversity and reduced SCFA. BMDM derived from high sugar fed mice were highly responsive to LPS. High sugar fed mice had increased susceptibility to colitis and pro-inflammatory cytokine concentrations. Oral acetate significantly attenuated colitis in mice by restoring permeability. In conclusion, short term exposure to a high sugar diet increases susceptibility to colitis by reducing short-chain fatty acids and increasing gut permeability.

## Introduction

Inflammatory bowel disease (IBD), and its two subtypes Crohn’s disease and ulcerative colitis are multifactorial in nature, developing due to a complex interplay between genetic, environmental, microbial, and immunologic factors^1^. Although the importance of diet in the development of IBD remains unclear, dietary factors do appear to play a role in either the triggering of disease flares or modulating disease phenotypes^2–4^. Of interest has been the parallel rise of both a ‘western diet’ (i.e. a diet that is high in fat and refined sugars) worldwide, and a corresponding geographic increase in the incidence of IBD, which continues to grow as a global health problem^5,6^.

Most experimental findings relating to the ‘western diet’ and IBD have focused on high-fat content alone or in combination with high-sugar; thus the isolated effects of dietary refined sugars on enteric microbial composition, luminal milieu, and susceptibility to colitis are not well understood^2^. Population-based studies have demonstrated an association between intake of refined sugar and artificial sweeteners and increased incidence of IBD^7–12^. In addition, dietary surveys indicate that ~10% of IBD patients feel that “sugary” foods trigger disease flare-ups and worsen severity of their symptoms^13,14^. Interestingly, the elevated incidence of IBD associated with a high-sugar diet can be reversed by a diet rich in fiber^8^. The protective mechanism of dietary fiber is thought to be due in part to the metabolism of carbohydrate polymers into short-chain fatty acids (SCFAs) by specific bacterial populations^15^. SCFAs, which include butyrate and acetate, are thought to play a role in IBD status which is supported by the fact that depressed levels of SCFAs have been described in the stool of IBD patients^16–18^.

In mouse models of IBD, the resident microbiota has been shown to influence disease severity and outcome and transfer of colitogenic microbial communities has been shown to alter colitis susceptibility in recipients^19,20^. Changes in gut microbial composition have also been associated with IBD; most notably decreases in the level of Firmicutes and/or Bacteroides and an increase in relative abundance of Proteobacteria have been described in numerous publications^21,22^. However, whether these alterations in gut microbiota are causal or occur due to the presence of inflammation in the intestine remains to be conclusively demonstrated^23^.

In this study, we aimed to assess the impact of a short-term dietary exposure to high sugar on colitis susceptibility in order to examine how daily fluctuations in diet may trigger disease flares in susceptible patients. Given the apparent relationship between a high-sugar diet and colitis we hypothesized that a diet high in refined sugar would elicit alterations in gut microbial metabolism and increase disease susceptibility.

## Methods

### Animal Model

**Figure 1:**
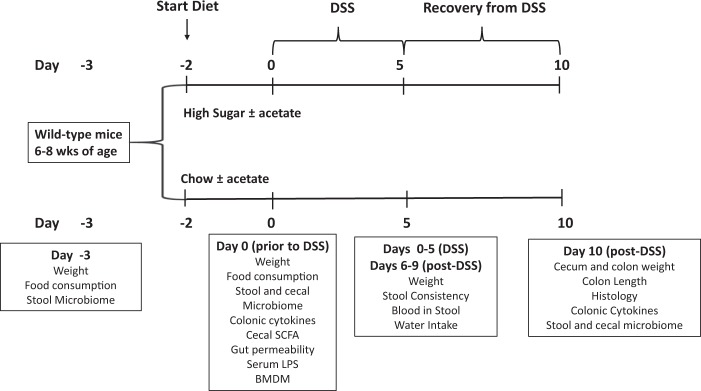
Experimental Design. Female mice on a 129S1/SvimJ background were raised on chow until 6–8 wks of age and then placed on either a chow diet (CH) (LabDiet 5001) or a high- sugar diet (HS) (50% Sucrose; Harlan Teklad AIN76A) (Day -2). After two days on the diet, mice were treated with dextran sodium sulfate (DSS 3%; MW 35–45,000 kDa; MP Biomedicals) added to their drinking water (Day 0). After five days, DSS was removed and replaced with regular drinking water for a further 5 days. Mice were sacrificed on the tenth day and tissues collected and snap frozen for further study. Weight and stools were collected on day -3 prior to the diet switch. The effect of two days on the diet (Day 0) on stool and cecal microbiome, colonic cytokine concentrations, cecal SCFA levels, gut permeability, and serum LPS was assessed. Body weight, stool consistency, and fecal occult blood (FOB) were measured daily from day 0 (start of DSS) to day 10. A parallel group of mice received acetate (NaAc:300 mM) in the drinking water throughout the experiment beginning at day -2 when the mice were switched to the different diets.

Wild-type mice on a 129S1/SvimJ background were raised at the University of Alberta and housed under conventional conditions. At 6–8 weeks of age, female mice were placed on either a chow diet (CH) (LabDiet 5001) or a high sugar diet (HS) (50% Sucrose; Harlan Teklad AIN76A) for a period of two days. Fiber content was 5% (cellulose) and 5.3% (crude fiber) in the HS and chow diets respectively. Mice from the same litter were randomized to separate cages and groups and each experiment was repeated twice. After two days on the different diets, mice were treated with a low concentration of dextran sodium sulfate (DSS 3%; MW 35–45,000 kDa; MP Biomedicals) added to their drinking water (Day 0). After five days, DSS was removed and replaced with regular drinking water. Mice were sacrificed on Day 10 and tissues collected and snap frozen for further study (Fig. 1). Body weight, stool consistency, and fecal occult blood (FOB) were measured daily. FOB positivity was determined using the Hemoccult (Beckman Coulter) test. A disease activity index (DAI) was used that included percentage weight loss, stool consistency, and blood in stool. Each parameter was measured on a scale of 0–4 for a total DAI ranging from 0–12. The weight and length of colon and the weight of the cecum was measured at the time of sacrifice. To determine the role of acetate, a parallel group of mice received acetate (NaAc:300 mM) in the drinking water throughout the experiment beginning at day -2 when the mice were switched to the different diets. All experiments were repeated in cohorts of mice from different litters. Animal use protocols were approved by the animal care committee at the University of Alberta and all experiments were carried out in accordance with the relevant guidelines and regulations.

### Histological injury

Colons were flushed with phosphate-buffered saline and immediately fixed in neutral buffered formalin (10%vol:vol). The fixed samples were processed with the use of standard paraffin-embedded histologic methods and hematoxylin and eosin staining. Disease scoring was based on a scoring method that included measurement of epithelial hyperplasia (0–3), enterocyte injury (0–3), and the presence of lymphocytes and neutrophils (0–4) in the lamina propria^24^. Total histologic score was calculated as the sum of the individual variables and had a maximum score of 10.

### Preparation of bone-marrow derived macrophages

Wild-type mice were placed on chow (n = 4) or high sugar diet (n = 4) for two days. At sacrifice, femurs and tibias from each mouse were removed and cleaned, ends of bones snipped and bone marrow flushed with 10 mL of magnesium and calcium free (MCF) phosphate buffered saline (PBS), then pooled for each mouse^25^. Pooled cells were centrifuged at 1000 rpm for 5 minutes and supernatant removed. The pellet was reconstituted in 2 mL of macrophage complete media. Bone marrow cells were enumerated using a Coulter counter and 2 × 10^6^ cells plated in 10 mL of macrophage complete media (MCM) which included Dulbecco’s modified eagle media (DMEM/F12), 10% heat inactivated fetal bovine serum (FBS), penicillin (100 U/ml), L-glutamine (10 mM), 100 ug/ml streptomycin and 20% macrophage colony-stimulating factor (M-CSF) obtained from L929 cell line. Cells were incubated at 37 °C and 5% CO_2_ for 7 days. On day 7, cells were washed with warm M-CSF PBS and then scraped and pooled into a 50 mL conical tube. Five mL of cold DMEM/F12-10 media was added to each conical and centrifuged for 10 minutes at 400 g and 4 °C. Cells were counted and 1 × 10^5^ cells plated into each well. Cells ± lipopolysaccharide (LPS;10 /µg/ml) were incubated at 37 °C and 5% CO_2_ incubator for 24 hours.

### Measurement of tissue cytokines

Snap frozen colonic tissue was homogenized in PBS containing 0.05% Tween 20. Homogenates were centrifuged at 9600 × g for 10 minutes. IFN-γ, IL-1β, IL-10, IL-12 p70, IL-2, IL-4, IL-5, KC/GRO (keratinocyte chemoattractant/human growth-regulated oncogene), IL-6, and TNF-α were evaluated using the Proinflammatory Panel 1 V-PLEX Mouse Kit (Meso Scale Discovery, Rockville, MD) as per manufacturer’s protocol. TGF-β was measured using ELISA duo set (R&D Systems, Inc., Minneapolis, MN). Cytokine levels were corrected for tissue weight.

### Microbiome analysis

Cecums were collected at sacrifice and immediately snap frozen in liquid nitrogen and stored at −80 °C until processed for either microbial composition or SCFA. Freshly voided stools were collected and frozen. For sequencing, DNA was extracted using AquaStool and cleaned using ethanol precipitation. Microbial composition was assessed using Illumina’s established 16S rRNA amplicon sequencing method and the MiSeq sequencing platform. Briefly, a segment of the V3 and V4 region of the 16S gene was amplified with gene specific primers (aligning to 341 bp and 805 bp in the gene) that also included an adapter sequence overhang: Bact_16s_ILL1_341mF 5-TCG TCG GCA GCG TCA GAT GTG TAT AAG AGA CAG CCT ACG GGN GGC WGC AG-3, Bact_16s_ILL1_805mR 5- GTC TCG TGG GCT CGG AGA TGT GTA TAA GAG ACA GGA CTA CHV GGG TAT CTA ATC C-3. This PCR reaction was cycled 25 times and the resulting reaction purified using bead-based clean-up followed by an 8 cycle PCR reaction using Illumina’s proprietary bar-coding primers that also align to the adapter sequence. After a second clean-up the bar-coded libraries were diluted, denatured, pooled and run using a V3 300 bp reagent cartridge on the MiSeq system. Bacterial composition was estimated using Quantitative Insights into Microbial Ecology (QIIME 1.9.1) pipelines^26^. QIIME was used to de-multiplex the barcoded reads and perform chimera filtering. Filtered sequence reads were grouped into OTUs at a sequence similarity level of 97%, which approximates species-level phylotypes. Taxonomy of the OTUs was assigned and sequences were aligned with RDP classifier and Pynast^27^. Alpha diversities of each microbial community were calculated using the Shannon diversity metric.

### Assessment of gut permeability

At sacrifice, a 10 cm portion of small intestine was excised distally from 10 cm below the ligament of Treitz and flushed with ice-cold phosphate-buffered saline (PBS). The intestinal segment was ligated at one end and a tube was inserted to add 400 µl of fluorescein isothiocyanate (FITC)/rhodamine dextran (40 µg/mL FITC-labelled 4 kDa dextran, and 40 µg/mL rhodamine labelled 70 kDa dextran in Hank’s Buffered Salt Solution). A ligature was applied as the tube was removed. The length and width of the segment was measured and then submerged in 10 mL of HBSS maintained at 37 °C and percolated with carbogen (95% O_2_, 5% CO_2_). Samples (100 µl) were collected from surrounding media at baseline and every ten minutes for thirty minutes. Fluorescence was measured using a SpectraMax M3 spectrophotometer (Molecular Devices, USA) fluorescence at 438ex/544em for FITC and once at 520ex/590em for rhodamine. A leakage ratio was calculated for each time point by normalizing dextran (µg) to the gut volume then dividing the 4 kDa dextran by 70 kDa dextran to obtain a ratio. Serum LPS measurement was carried out using the Pyrochrome Limulus Amoebocyte Lysate (LAL) assay according to manufacturer’s instructions (Associates of Cape Cod Incorporated).

### Measurements of short-chain fatty acids (SCFA)

The concentrations of SCFA in cecal contents were determined using gas chromatography. Stool (0.2 g) was homogenized in 800 uL of 0.1 N hydrochloric acid. Phosphoric acid (200 µl of 25%) was then added and the sample centrifiuged at 3 000 × g for ten minutes. Supernatant was added to internal standard solution (150 mg of 4-methyl-valeric acid, S381810, Sigma-Aldrich) and 5% phosphoric acid in a glass chromatography tube, mixed well, and kept at room temperature for 30 min. The supernatant was analyzed for SCFA using a Varian model 3400 Gas Chromatograph (Varian, Walnut Creek, CA) with a Stabilwax-DA column (30-m × 0.25-mm i.d.; Restek, Bellefonte, PA). A flame-ionization detector was used with an injector temperature of 170 °C and a detector temperature of 190 °C.

### Statistical analysis

All data is presented as the mean ± SEM. Statistical analysis was performed using STATA v13.1. Differences between group means were evaluated by using one-way analysis of variance with Tukey post hoc test to correct for multiple comparisons. White’s nonparametric t-test was used to compare microbial communities between groups, with Benjamini-Hochberg test applied to assess for false discovery rate. Principal component analysis (PCA) plots of bacterial populations were created using Metaboanalyst 3.0. Significance was defined as p < 0.05.

## Results

### High-sugar diet impairs barrier function and enhances susceptibility to DSS-induced colitis

**Figure 2:**
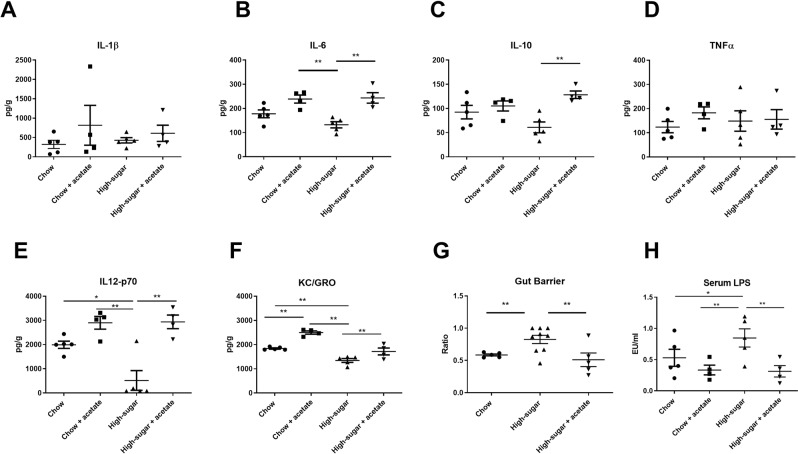
Effects of a high sugar diet and acetate supplementation on colonic tissue cytokines and barrier function. Colonic tissue concentrations of IL-1β (**A**), IL-6 (**B**), IL-10 (**C**), TNFα (**D**), IL-12p70 (**E**), and KC/GRO (**F**). Two days of high-sugar diet significantly decreased IL-12p70 and KC/GRO levels compared to chow fed mice. Oral acetate supplementation in a high-sugar diet elevated the concentration of IL-6 (**B**), IL-10 (**C**), and IL-12p70 (**E**). Barrier function following two days of a high-sugar diet as measured by the ratio of 70 kDa/4 kDa dextran movement through intestinal loops (**G**) and serum LPS levels (**H**). Mice on a high-sugar diet had an increased ratio representing a decrease in barrier function compared to chow-fed mice and increased serum LPS compared with chow, chow + acetate, and HS + acetate fed mice. Acetate supplementation normalized intestinal barrier function. Data is shown as mean ± SEM. Chow: n = 5; HS: n = 5; Chow + acetate: n = 4; HS + acetate: n = 4. **p < 0.01; *p < 0.05.

**Figure 3:**
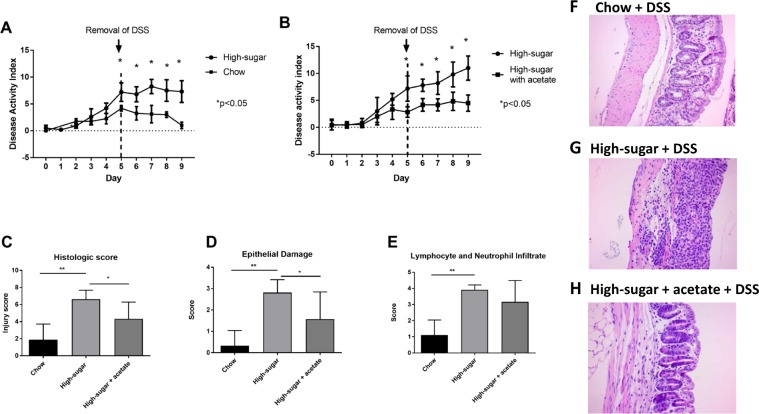
Mice on a high sugar diet had increased susceptibility to colitis and delayed recovery. (**A**) Disease activity index (DAI) demonstrating an increase in disease severity and lack of repair following removal of DSS in mice consuming a high-sugar diet (*p < 0.05). (**B**) Mice on the high-sugar diet receiving oral acetate had decreased disease severity as evidenced by decreased DAI and enhanced recovery following removal of DSS (*p < 0.05). Mice on the high-sugar diet had a significant increase in total histological score (**C**) (0–10), increased epithelial damage (**D**) (0–4), and increased lymphocyte and neutrophilic infiltration (**E**) (0–4) compared with chow fed mice. Oral acetate significantly improved total histological score and reduced epithelial damage. Representative photomicrographs of hematoxylin-eosin stained sections of colon (original magnification 200x) from chow (**F**), high sugar (**G**) and high-sugar + acetate (**H**) fed mice. Data is shown as mean ± SEM. Chow: n = 5; HS: n = 5; Chow + acetate: n = 4; HS + acetate: n = 4. **p < 0.01; *p < 0.05.

**Figure 4:**
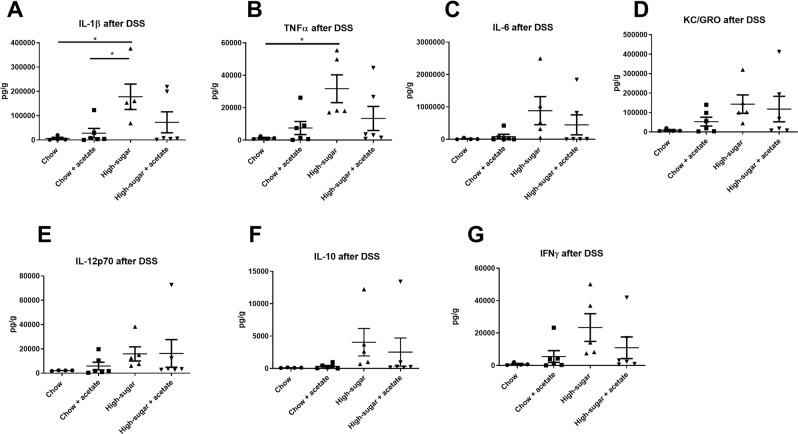
Cytokine levels in colonic tissue following DSS treatment. IL-1β (**A**) and TNFα (**B**) were increased in high-sugar fed mice compared with chow fed mice following DSS administration. Data is shown as Mean ± SEM. Chow: n = 4; HS: n = 6; Chow + acetate: n = 6; HS + acetate: n = 6. *p < 0.05.

Mice on a high sugar diet (HS; n = 5) for two days had no changes in weight, caloric consumption, or general activity level compared with mice which remained on chow diet (data not shown). There were also no changes seen in colonic tissue levels of IL-1β (Fig. 2A), IL-6 (Fig. 2B), IL-10 (Fig. 2C), or TNFα (Fig. 2D). However, a HS diet did lead to a significant depression in the colonic concentration of IL-12p70 (Fig. 2E) and the neutrophil chemoattractant, KC/GRO (Fig. 2F). Mice on the HS diet also demonstrated a significant increase in gut permeability as assessed directly in intestinal loops (Fig. 2G) (p < 0.01) and indirectly by increased levels of LPS in the serum (Fig. 2H) (p < 0.05). There were no differences in levels of IL-2, IL-4, IL-5, IFNγ or TGFβ (data not shown). To determine if consuming a HS diet over two days altered disease susceptibility, mice were given dextran sodium sulfate (DSS). As seen in Fig. 3A, HS fed mice had an increased severity of disease by Day 5 and upon removal of DSS, failed to recover while chow fed mice showed a complete recovery (Fig. 3A). Further physical evidence of increased colitis was manifested as a shortened colon in the HS fed mice at the time of sacrifice (Table 1). Histological analysis showed an increased total histological injury in HS fed mice (Fig. 3C), increased epithelial damage (Fig. 3D) and increased lymphocytic and neutrophilic infiltration into the lamina propria (Fig. 3E). Representative histological images of effects of DSS treatment in chow and HS-fed mice are shown in Fig. 3F,G respectively. Increased severity of colitis was also evidenced by increased tissue levels of IL-1β (Fig. 4A) and TNFα (Fig. 4B) (p < 0.05) following DSS administration. There were no significant changes in IL-6 (Fig. 4C), KC/GRO (Fig. 4D), IL-12p70 (Fig. 4E), IL-10 (Fig. 4F) or IFNγ (Fig. 4G).

**Table 1:** Colon length and tissue weights in chow and high sugar fed mice following DSS.

Group	Treatment	Cecum Weight (g)	Length (cm)	Weight (g)	Weight/Length (g/cm)
Chow	None (n = 5)	0.18 ± 0.03^a^	8.6 ± 0.9^a^	0.18 ± 0.02^b^	0.021 ± 0.003^a^
	DSS (n = 5)	0.15 ± 0.05^ab^	6.8 ± 0.5^b^	0.23 ± 0.04^a^	0.034 ± 0.002^b^
	Acetate (n = 5)	0.19 ± 0.05^a^	8.3 ± 0.5^a^	0.22 ± 0.02^a^	0.026 ± 0.003^ab^
	Acetate + DSS (n = 4)	0.15 ± 0.03^ab^	7.1 ± 0.9^b^	0.23 ± 0.02^a^	0.032 ± 0.001^b^
High Sugar	None (n = 5)	0.16 ± 0.05^a^	8.0 ± 0.5^a^	0.19 ± 0.02^b^	0.023 ± 0.004^a^
	DSS (n = 5)	0.08 ± 0.02^b^	5.2 ± 0.4^c^	0.17 ± 0.01^b^	0.033 ± 0.002^b^
	Acetate (n = 4)	0.14 ± 0.03^ab^	7.8 ± 0.5^a^	0.18 ± 0.02^b^	0.023 ± 0.002^a^
	Acetate + DSS (n = 4)	0.10 ± 0.01^b^	6.2 ± 0.4^b^	0.18 ± 0.01^b^	0.029 ± 0.001^ab^

Values are means ± SEM. Labeled means in a row without a common letter differ. p < 0.05.

### High- sugar diet induces changes in bone-marrow derived monocytes (BMDM)

**Figure 5:**
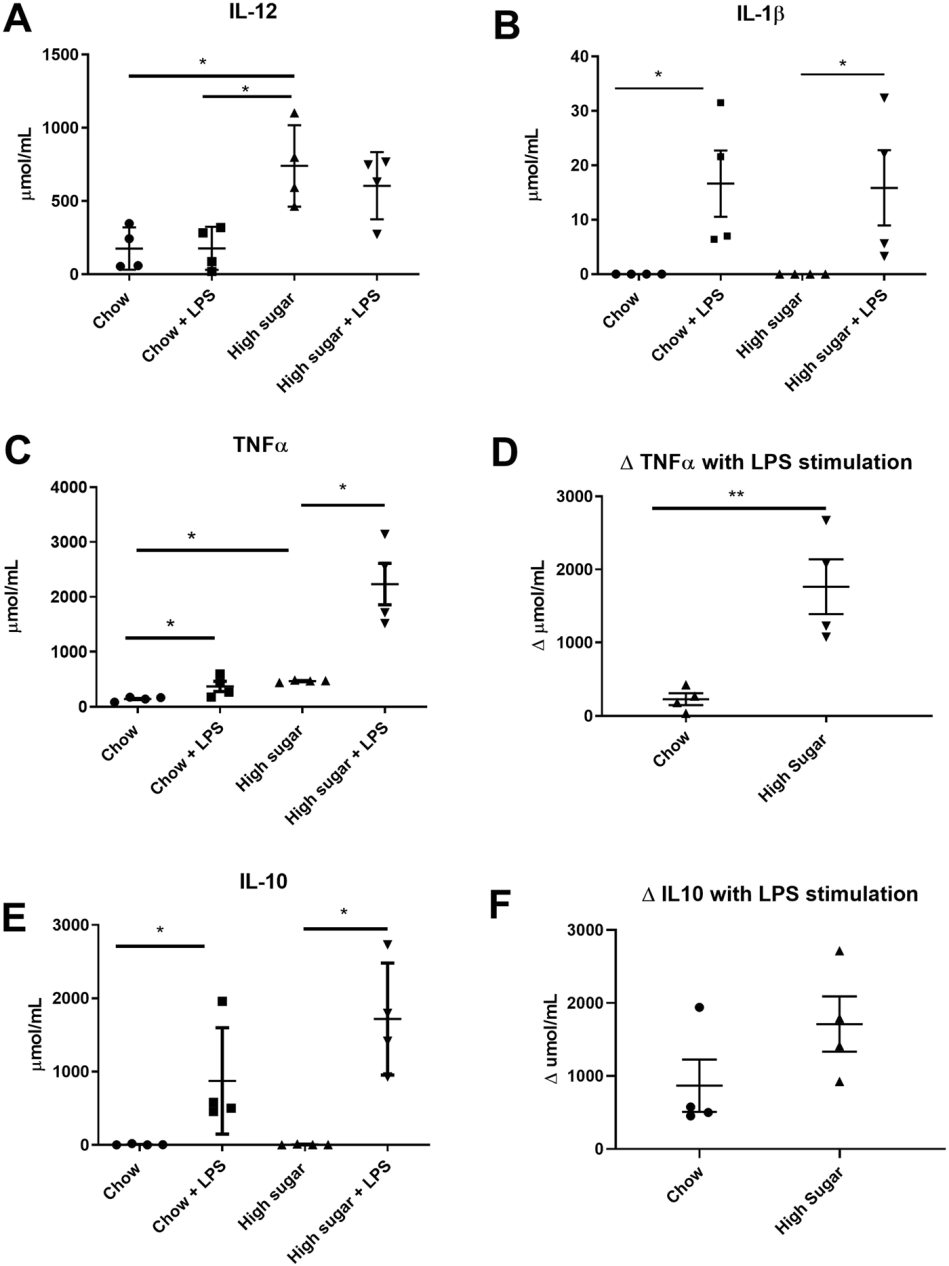
Cytokine secretion from bone-marrow derived monocytes (BMDM). BMDM generated from chow and high sugar fed mice were incubated ± lipopolysaccharide for 24 hours. BMDM from high sugar fed mice had increased basal secretion of IL-12p70 (**A**) and TNFα (**C**). BMDM generated from both chow and high-sugar fed mice responded to LPS with increased secretion of IL-1β (**B**), TNFα (**C**), and IL-10 (**E**). ΔTNFα secretion in response to LPS stimulation was higher in BMDM from high sugar fed mice compared with ΔTNFα secretion in BMDM from chow fed mice. (**D**). There was no difference in ΔIL-10 secretion to LPS stimulation between the two groups. Data is shown as mean ± SEM. Chow: n = 4; HS: n = 4. **p < 0.01; *p < 0.05.

The DSS model is characterized by damage to enterocytes and invasion of luminal bacteria; this then induces a massive infiltration of neutrophils and blood monocytes into the lamina propria which both amplify and then effectively resolve inflammation^28^. A systemic priming of blood monocytes by cytokines or other factors can effectively result in heighted pro-inflammatory cytokine responses by monocytes to bacteria products and an exacerbation of inflammation^29^. In that HS-fed mice showed increased severity and lack of resolution of inflammation, we examined bone-marrow derived monocytes to determine the responsiveness of these systemic immune cells to bacterial products following 2 days on the HS diet. BMDMs derived from HS fed mice exhibited enhanced basal secretion of IL-12p70 (Fig. 5A) and TNFα (Fig. 5C) compared to BMDMs from chow fed mice. There was no difference in basal IL-1β (Fig. 5B) or IL-10 (Fig. 5E) secretion between the two groups. When treated with LPS, all BMDMs increased secretion of IL-1β (Fig. 5B), TNFα (Fig. 5C) and IL-10 (Fig. 5E). BMDMs from HS fed mice released significantly more TNFα when stimulated with LPS compared with BMDM from chow-fed mice (Fig. 5D).

### High-sugar diet leads to rapid microbial dysbiosis

**Figure 6:**
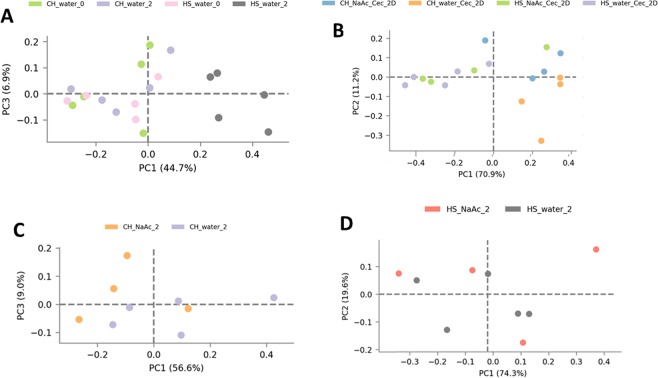
High-sugar diet induces marked microbial dysbiosis. PCA plot of the microbial populations in stool samples from chow and high-sugar fed mice at day -3 (Chow: green, CH_water_0; HS: pink, HS_water_0) and after two days on the diet (Chow: purple, CH_water_2; HS: black, HS_water_2) (**A**). At day -3, all samples clustered together as expected. Following 2 days of a high-sugar diet (HS: black, HS_water_2), stool samples from mice on the high-sugar diet clustered separately from the chow-fed mice (Chow: purple, CH_water_2). Cecal samples in chow and high sugar fed mice after two days are shown in (**B**). Samples from the chow fed mice (Chow: orange, CH_water_Cec_2D) and the chow fed mice receiving acetate (Chow + Acetate: blue, CH_NaAc_Cec_2D) clustered together while stools from the high-sugar fed (HS: purple, HS_water_Cec_2D) and the high-sugar + acetate (HS + acetate: green, HS_NaAc_Cec_2D) mice also clustered together but apart from the samples from chow fed mice. Stool samples taken after two days on the diet from chow and chow + acetate mice are shown in (**C**). Samples from chow fed (Chow: purple, CH-water_2) and chow fed + acetate (Chow + Acetate: orange, CH_NaAc_2) clustered together. Stool samples taken after two days on the diet in high-sugar and high-sugar + acetate mice are shown in (**D**). Stool samples from high-sugar (HS: black, HS_water_2) and high-sugar + acetate (HS + Acetate: orange, HS_NaAc_2) fed mice also clustered together indicating that acetate did not alter the microbial changes induced by the high-sugar diet. Chow: n = 5; HS: n = 5; Chow + acetate: n = 4; HS + acetate: n = 4.

**Figure 7:**
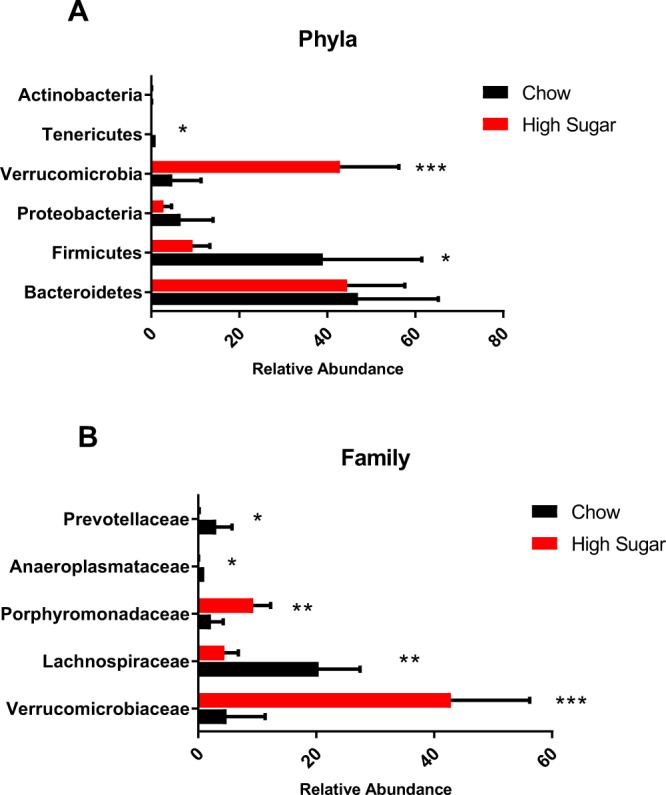
Effects of high-sugar diet on microbiota at the phyla and family levels. At the Phyla level, Verrucomicrobia was elevated in high sugar fed mice (p < 0.01, FDR < 0.01), while Firmicutes and Tenericutes were depleted (p = 0.02, FDR < 0.04) (**A**). At the family level, Lachnospiraceae, Prevotellaceae, and Anaeroplasmataceae were significantly decreased by a high-sugar diet (p < 0.01, FDR < 0.02) while Verrucomicrobiaceae and Porphyromonadaceae were significantly elevated (p < 0.01, FDR  < 0.02) (**B**). Data is shown as mean ± SEM of relative abundance. Chow: n = 5; HS: n = 5.

In that alterations in gut microbial composition have been linked with increased susceptibility to colitis^30^, we analyzed microbiota in stools and cecal contents prior to and following the diet switch. Figure 6A shows stools taken from individual mice prior to and following two days on a HS or chow diet. Stools taken from mice prior to diet change clustered together. Mice remaining on the chow diet for 2 days also clustered with the baseline samples. However, stools from mice that switched to the HS diet clustered apart and separately from the baseline samples. Figure 6B shows that cecum samples from chow and HS-fed mice also clustered apart. Complete microbial profiles are shown in Supplementary Fig. 1 and relative abundance values in Supplementary Table 1. At the phyla level, Verrucomicrobia was found to be elevated in HS fed mice (p < 0.01, FDR < 0.01), while Firmicutes and Tenericutes were depleted (p = 0.02, FDR < 0.04) (Fig. 7A). At the family level, Lachnospiraceae, Anaeroplasmataceae and Prevotellaceae were significantly decreased by a HS diet (p < 0.01, FDR < 0.02) while Verrucomicrobiaceae and Porphyromonadaceae were significantly elevated (p < 0.01, FDR < 0.02) (Fig. 7B). HS fed mice also demonstrated an overall loss of α-diversity compared to chow-fed mice as measured by the Shannon index (Chow: 6.5 ± 0.4; HS: 5.1 ± 0.3; p < 0.02).

### Acetate partially reversed the enhanced susceptibility to colitis induced by a high-sugar diet

**Figure 8:**
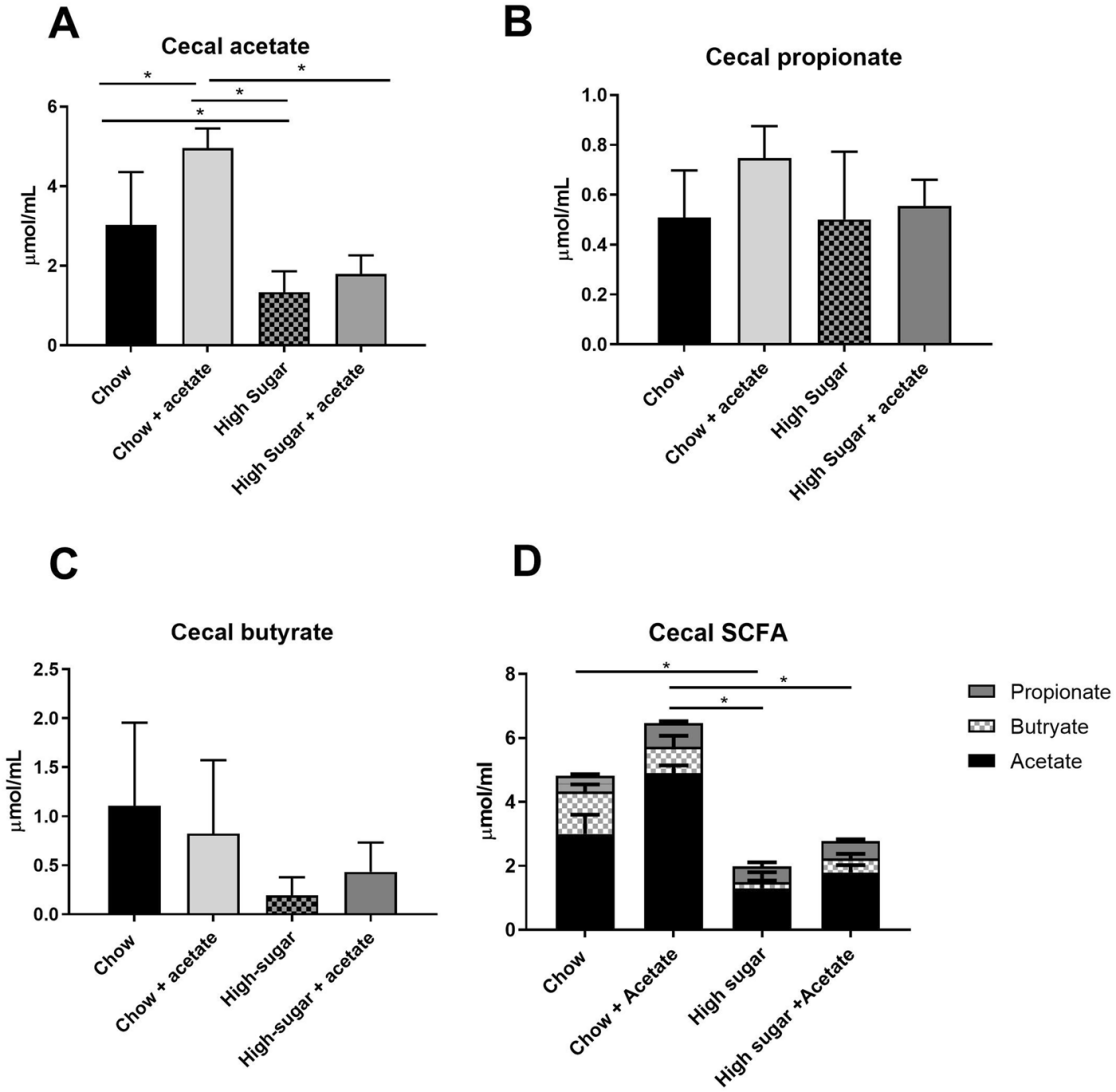
Short-chain fatty acids are depleted in mice fed a high-sugar diet. Concentrations of cecal acetate (**A**), propionate (**B**), butyrate (**C**), and total SCFA after two days on the diet. Cecal acetate and total concentrations of SCFA were significantly reduced in high sugar fed mice. Oral acetate supplementation increased cecal levels of acetate in chow fed mice (**A**). Data is shown as mean ± SEM. Chow: n = 5; HS: n = 5; Chow + acetate: n = 4; HS + acetate: n = 4. *p < 0.05.

The microbial changes induced by two days on a HS diet were associated with significant changes in SCFA concentrations in the cecum. In particular, acetate was found to be significantly depleted in cecal contents compared to chow fed mice (p < 0.05) (Fig. 8A). There were no significant changes in levels of cecal propionate (Fig. 8B) or butyrate (Fig. 8C). Total levels of cecal SCFA were also reduced in the HS fed mice (Fig. 8D) (p < 0.05). Given the significant reduction in cecal levels of acetate following two days of a HS diet, we hypothesized that a loss of luminal acetate was critical in enhancing disease susceptibility. To test this hypothesis, we supplemented mice with acetate in the drinking water prior to DSS. There was no significant difference in drinking water consumption between acetate-treated and nontreated mice (data not shown). The addition of acetate modulated the inflammatory milieu of the colon prior to the introduction of DSS in HS-fed mice, significantly increasing the concentrations of IL-6 (Fig. 2B), IL-10 (Fig. 2C), and IL-12p70 (Fig. 2E). Chow fed mice receiving acetate showed a significant increase in KC/GRO levels compared with chow fed alone (Fig. 2F). Acetate increased the concentration of IL-12p70 to a greater extent than IL-10 compared to the baseline values seen in HS fed mice (Elevation of IL-12: 463% (108), Elevation of IL-10: 111% (25) p < 0.03). In addition, acetate prevented the increase in intestinal permeability seen in the HS fed mice (Fig. 2G) and reduced levels of serum LPS (Fig. 2H). Compared with mice on the HS diet, mice on the HS diet supplemented with acetate had decreased severity of DSS-induced colitis and enhanced repair as evidenced by reduced DAI (Fig. 3B), reduced total histological score (Fig. 3C), reduced epithelial damage (Fig. 3E) and increased colon length (Table 1).

### Acetate did not alter intestinal microbial composition

The addition of acetate to the drinking water of mice had no significant effect on microbial community composition in either stool (Chow: Fig. 6C; HS: Fig. 6D) or cecum (Fig. 6B) of chow and HS fed mice. (Supplementary Fig. 1). Interestingly, while supplementation of chow-fed mice with acetate did significantly elevate the level of acetate in the cecum (Fig. 8A), this was not mirrored in the HS fed group. Levels of cecal propionate or butyrate were not altered by either diet or acetate supplementation (Fig. 8).

## Discussion

In this study we demonstrate that a two-day exposure to a high-sugar diet rapidly alters gut microbial composition, depletes short chain fatty acids and increases susceptibility to chemically induced colitis. This effect was significantly attenuated through the supplementation of acetate independently of changes in microbial community composition. These findings are consistent with recent literature purporting the risks of a high-sugar diet in the triggering and perpetuation of inflammatory bowel diseases and the protective role of SCFA^8,15,31^.

The dramatic effect of just two days of a high-sugar diet on microbial function and populations may shed light on the role of diet in triggering disease flares in patients with inflammatory bowel disease. An especially meaningful change in this study included the loss of important SCFA producing microbes belonging to the *Lachnospiraceae* family. This family is capable of producing numerous SCFAs, including butyrate and acetate^32^. A high sugar diet was also associated with an overall decrease in microbial α-diversity compared to chow diet. Community diversity and the interactions between different microbes are crucial in the production of SCFAs^33,34^. Therefore, the loss of individual microbial groups important in the creation of SCFAs, alongside a loss in overall community α-diversity, may be responsible for the decline in luminal SCFAs seen in a high-sugar diet. Interestingly, some artificial sweeteners have also been shown to alter gut microbiota and influence host physiological responses^35–37^. A study by Suez *et al*.^36^ showed that consumption of saccharin for 5 days in healthy human volunteers resulted in decreased glycemic responses that correlated with alterations in gut microbiota. In an animal model of CD-like ileitis, a 6-week intake of Splenda (sucralose maltodextrin) caused a bloom of *Proteobacteria* and gut dysbiosis with increased evidence of local gut inflammation^37^. It remains to be shown however, if short-term intake of sweeteners also increases susceptibility to colitis.

The loss of intestinal barrier function in the mice fed the high sugar diet could have been due to either a direct effect of high levels of luminal sucrose or alternatively linked with a decrease in SCFA production. SCFA, including acetate, propionate, and butyrate, are produced by gut microbial fermentation along the entire intestinal tract including the small intestine^38,39^. SCFA regulate gut immune and barrier function, as well as having a role in epithelial cell proliferation, differentiation, and apoptosis. SCFA also promote nutrient absorption, lipid metabolism, mucin production, and expression of antimicrobial peptides^31^. The importance of acetate in gut health was reinforced by the improvement in colitis susceptibility following the supplementation of acetate. Acetate did not elicit any significant changes in bacterial community composition or increase levels of butyrate but was able to independently enhance the ability of high sugar fed mice to recover from an intestinal chemical insult. Several studies have previously shown that luminal acetate induces protective barrier effects in the intestine both directly through effects on epithelial cells and indirectly through effects on immune cells^40–42^. In addition, oral acetate has previously been shown to reduce severity of DSS colitis when given prior to DSS^43,44^. In our study, the loss of small intestinal barrier function that occurred during high sugar feeding was prevented by the provision of acetate in the drinking water. This would suggest that the increased gut permeability was not occurring due to high levels of luminal glucose, but rather a lack of SCFA production. Interestingly the provision of oral acetate did not result in an increase in levels of cecal acetate in the mice fed the high sugar diet. However, acetate is both absorbed in the small intestine and used as cross feeding by many microbial species; thus, a lack of increase in the cecum was likely due to utilization in the upper gut.

In addition to increases in gut permeability prior to DSS treatment, high sugar fed mice also exhibited decreased colonic tissue levels of IL12p70 and KC-GRO suggesting dietary-induced changes in innate immune function in the colon. In contrast, following DSS, enhanced tissue levels of TNFα and IL-1β were seen in the high sugar fed mice. Interactions and crosstalk between resident intestinal macrophages, T cells, dendritic cells, epithelial cells and innate lymphoid cells are important in maintaining tissue homeostasis and in coordinating responses to injury. The DSS model of colitis is characterized by damage to enterocytes and invasion of luminal bacteria followed by an influx of neutrophils and blood monocytes from the systemic circulation into the lamina propria^29^. This influx of blood monocytes is critical both for the initial response to bacterial invasion and for the resolution of tissue damage. Depletion of monocytes has been shown to reduce disease severity in acute models of inflammation, including in the DSS model, indicating their importance in the pathophysiology of acute inflammation^45^. The reduced colonic tissue levels of IL12-p70 and KC-GRO prior to DSS treatment indicated dietary-induced localized effects on colonic immune function. In order to determine if consuming a diet high in sugar had systemic as well as localized effects on immune function, we examined the phenotype of isolated bone-marrow derived monocytes (BMDM) from high sugar fed mice before treatment with DSS. An interesting finding in this study was that BMDM from high sugar fed mice were more responsive to inflammatory stimuli compared to BMDM from chow fed mice and produced higher amounts of TNFα and IL-12p70 under basal conditions. It is possible that this altered phenotype of BMDM occurred due to the increased levels of LPS in the serum of high sugar mice as previous studies have suggested that exposure of blood monocytes to signals in the systemic circulation such as inflammatory cytokines or LPS can result in monocytes that are primed to release higher levels of pro-inflammatory cytokines^29^. TNFα and IL-12p70 are key markers of an inflammatory or M1 macrophage phenotype^46,47^, suggesting that BMDM from the high sugar fed mice were already primed and differentiated towards an inflammatory M1 phenotype prior to their influx into the lamina propria. This influx of enhanced inflammatory monocytes likely contributed to the increased tissue levels of TNFα and IL-1β seen in the high sugar fed mice and contributed to the increased severity of inflammation.

In conclusion, this paper provides mechanistic insight into the epidemiologic findings implicating a high-sugar diet in the triggering and perpetuation of inflammation in patients with inflammatory bowel disease. The results show that a short-term diet high in sugar results in an enhanced intestinal permeability coupled with a more inflammatory monocyte phenotype that results in an increased susceptibility to colonic insults and a lack of ability to repair damage. The findings that acetate alone substantially alleviates the deleterious effects of a high-sugar diet adds to the body of evidence suggesting that SCFAs play a crucial role in the response of the gut to insults and tissue repair.

## Supplementary information

Supplementary Information
