# Supplementary material for: A high-sugar diet rapidly enhances susceptibility to colitis via depletion of luminal short-chain fatty acids in mice
Source: Sci Rep. 2019 Aug 23;9:12294. doi: 10.1038/s41598-019-48749-2 (PMC6707253; doi:10.1038/s41598-019-48749-2)
Supplement: Supplementary file 1 — Supplementary Information [file 41598_2019_48749_MOESM1_ESM.pdf]

A high-sugar diet rapidly enhances susceptibility to colitis via depletion of luminal short-chain fatty acids in mice

Michael Laffin MD[1,3*] Robert Fedorak, BSc[2,3*], Aiden Zalasky, BSc[2,3], Heekuk Park PhD[2,3], Amanpreet Gill [2,3], Ambika Agrawal[2,3], Ammar Keshteli[2,3], Naomi Hotte[2,3], Karen L. Madsen[2,3+]

[1]Department of Surgery, University of Alberta; [2]Department of Medicine, University of Alberta, Edmonton, Alberta Canada T6G 2E1; [3]The Centre of Excellence for Gastrointestinal Inflammation and Immunity Research (CEGIIR), University of Alberta, Edmonton, Alberta, Canada T6G 2E1

Supplementary Information

Supplemental Table 1. Relative abundances of fecal microbes in chow and high-sugar fed mice after two days of diet

Supplemental Figure 1. Relative abundances of bacteria at the phyla and family levels in stool samples from individual mice at day -3 (prior to start of diet) and at day 0 (after 2 days on diet ± acetate and prior to DSS). Cecum samples were analyzed in individual mice on Day 0 (2 days on diet ± acetate and prior to DSS). HS: high sugar. CH: chow. NaAc: Sodium acetate. Cec: Cecum.

Supplemental Table 1. Relative abundances of fecal microbes in chow and high-sugar fed mice after two days of diet

	Chow diet	SD	High-sugar diet	SD	p-values	FDR
**Phyla**						
Verrucomicrobia	4.824576	6.52518	42.85236	13.3653	**0.000857**	**0.006**
Tenericutes	1.002815	0.710155	0.025408	0.021829	**0.011857**	**0.0415**
Firmicutes	39.83634	22.58539	9.40704	3.90678	**0.019571**	**0.045667**
Proteobacteria	6.764601	7.387729	2.850808	1.872713	0.362	0.6335
Bacteroidetes	47.18191	18.20676	44.52112	13.15364	0.778857	0.908667
Actinobacteria	0.050156	0.035119	0.048471	0.032134	0.926143	0.926143
**Class**						
Verrucomicrobiae	4.824576	6.52518	42.85236	13.3653	**0.00075**	**0.009**
Mollicutes	1.002815	0.710155	0.025408	0.021829	**0.012**	0.072
Clostridia	39.30348	22.5447	9.278293	3.872021	**0.021417**	0.085667
Erysipelotrichi	0.002467	0.003022	0.018016	0.014712	0.052167	0.1565
Bacilli	0.530391	0.472922	0.11073	0.071567	0.107583	0.2582
Gammaproteobacteria	0.051919	0.056161	0.214467	0.20332	0.162083	0.324167
Actinobacteria	0.001253	0.002505	0.020935	0.030072	0.252083	0.432143
Alphaproteobacteria	6.091873	7.204506	1.810717	1.557043	0.294917	0.442375
Coriobacteriia	0.048903	0.032907	0.027537	0.017513	0.29975	0.399667
Betaproteobacteria	0.620809	0.209882	0.825625	0.350862	0.370583	0.4447
Bacteroidia	47.18191	18.20676	44.52112	13.15364	0.79625	0.79625
**Order**						
Verrucomicrobiales	4.824576	6.52518	42.85236	13.3653	**0.0005**	**0.0075**
Anaeroplasmatales	1.002815	0.710155	0.025408	0.021829	**0.010167**	0.07625
Clostridiales	39.30348	22.5447	9.278293	3.872021	**0.02275**	0.11375
Erysipelotrichales	0.002467	0.003022	0.018016	0.014712	0.054667	0.205
Lactobacillales	0.529215	0.47353	0.11073	0.071567	0.108417	0.32525
Enterobacteriales	0.050666	0.057088	0.214467	0.20332	0.159833	0.399583
Bifidobacteriales	0.001253	0.002505	0.020935	0.030072	0.248	0.531429
RF32	6.091873	7.204506	1.810717	1.557043	0.293333	0.55
Coriobacteriales	0.048903	0.032907	0.027537	0.017513	0.297417	0.495694
Burkholderiales	0.620809	0.209882	0.825625	0.350862	0.364583	0.546875
Turicibacterales	0.001176	0.002352	0	0	0.498459	0.679717
Pseudomonadales	0.001253	0.002505	0	0	0.498459	0.623074
Bacteroidales	47.18191	18.20676	44.52112	13.15364	0.789917	0.846339
**Family**						
Verrucomicrobiaceae	4.824576	6.52518	42.85236	13.3653	**0.000524**	**0.014667**
Lachnospiraceae	20.41525	7.040255	4.445972	2.339978	**0.001048**	**0.014667**
Porphyromonadaceae	2.152951	2.077367	9.31845	2.955981	**0.001286**	**0.012**
Anaeroplasmataceae	1.002815	0.710155	0.025408	0.021829	**0.013286**	0.093
Prevotellaceae	3.040375	2.662938	0.067972	0.071616	**0.041143**	0.2304

Erysipelotrichaceae	0.002467	0.003022	0.018016	0.014712	0.054095	0.252444
Bacteroidaceae	7.07656	2.68484	17.34949	9.632654	0.062143	0.248571
S24-7	34.59883	15.50786	17.42222	7.092572	0.068762	0.240667
Lactobacillaceae	0.527982	0.474544	0.108251	0.074237	0.112667	0.350519
Enterobacteriaceae	0.050666	0.057088	0.214467	0.20332	0.155619	0.396121
Bifidobacteriaceae	0.001253	0.002505	0.020935	0.030072	0.242286	0.565333
Coriobacteriaceae	0.048903	0.032907	0.027537	0.017513	0.296524	0.593048
Alcaligenaceae	0.620809	0.209882	0.825625	0.350862	0.364714	0.63825
Ruminococcaceae	2.940501	2.407005	1.611508	1.507888	0.405095	0.667216
Clostridiaceae	0.006072	0.005473	0.013298	0.016477	0.446429	0.694444
Turicibacteraceae	0.001176	0.002352	0	0	0.498459	0.734571
Pseudomonadaceae	0.001253	0.002505	0	0	0.498459	0.697843
Rikenellaceae	0.290563	0.208413	0.353299	0.430385	0.778429	0.990727
Dehalobacteriaceae	0.08778	0.085155	0.087963	0.083495	0.995571	1.212
Enterococcaceae	0.001232	0.002465	0.00248	0.004959	1	1.166667

Microbial comparisons performed using White’s nonparametric t-test with false-discovery rate correction using the Benjamini-Hochberg method

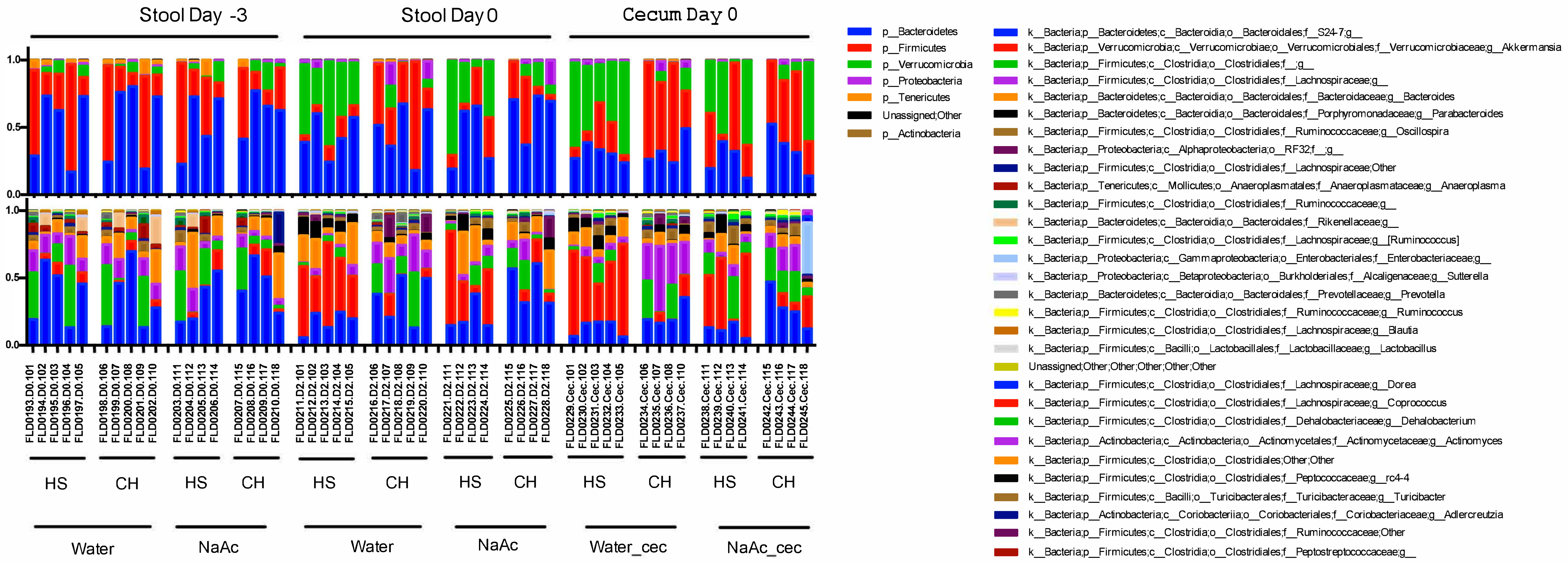
Stool Day -3
Stool Day 0
Cecum Day 0
1.0
0.5
0.0
FLD0193.D0.101
FLD0194.D0.102
FLD0195.D0.103
FLD0196.D0.104
FLD0197.D0.105
FLD0198.D0.106
FLD0199.D0.107
FLD0200.D0.108
FLD0201.D0.109
FLD0202.D0.110
FLD0203.D0.111
FLD0204.D0.112
FLD0205.D0.113
FLD0206.D0.114
FLD0207.D0.115
FLD0208.D0.116
FLD0209.D0.117
FLD0210.D0.118
FLD0211.D2.101
FLD0212.D2.102
FLD0213.D2.103
FLD0214.D2.104
FLD0215.D2.105
FLD0216.D2.106
FLD0217.D2.107
FLD0218.D2.108
FLD0219.D2.109
FLD0220.D2.110
FLD0221.D2.111
FLD0222.D2.112
FLD0223.D2.113
FLD0224.D2.114
FLD0225.D2.115
FLD0226.D2.116
FLD0227.D2.117
FLD0228.D2.118
FLD0229.Cec.101
FLD0230.Cec.102
FLD0231.Cec.103
FLD0232.Cec.104
FLD0233.Cec.105
FLD0234.Cec.106
FLD0235.Cec.107
FLD0236.Cec.108
FLD0237.Cec.110
FLD0238.Cec.111
FLD0239.Cec.112
FLD0240.Cec.113
FLD0241.Cec.114
FLD0242.Cec.115
FLD0243.Cec.116
FLD0244.Cec.117
FLD0245.Cec.118
HS
CH
HS
CH
HS
CH
HS
CH
HS
CH
HS
CH
Water
NaAc
Water
NaAc
Water_cec
NaAc_cec
p__Bacteroidetes
p__Firmicutes
p__Verrucomicrobia
p__Proteobacteria
p__Tenericutes
Unassigned;Other
p__Actinobacteria
k__Bacteria;p__Bacteroidetes;c__Bacteroidia;o__Bacteroidales;f__S24-7;g__
k__Bacteria;p__Verrucomicrobia;c__Verrucomicrobiae;o__Verrucomicrobiales;f__Verrucomicrobiaceae;g__Akkermansia
k__Bacteria;p__Firmicutes;c__Clostridia;o__Clostridiales;f__;g__
k__Bacteria;p__Firmicutes;c__Clostridia;o__Clostridiales;f__Lachnospiraceae;g__
k__Bacteria;p__Bacteroidetes;c__Bacteroidia;o__Bacteroidales;f__Bacteroidaceae;g__Bacteroides
k__Bacteria;p__Bacteroidetes;c__Bacteroidia;o__Bacteroidales;f__Porphyromonadaceae;g__Parabacteroides
k__Bacteria;p__Firmicutes;c__Clostridia;o__Clostridiales;f__Ruminococcaceae;g__Oscillospira
k__Bacteria;p__Proteobacteria;c__Alphaproteobacteria;o__RF32;f__;g__
k__Bacteria;p__Firmicutes;c__Clostridia;o__Clostridiales;f__Lachnospiraceae;Other
k__Bacteria;p__Tenericutes;c__Mollicutes;o__Anaeroplasmatales;f__Anaeroplasmataceae;g__Anaeroplasma
k__Bacteria;p__Firmicutes;c__Clostridia;o__Clostridiales;f__Ruminococcaceae;g__
k__Bacteria;p__Bacteroidetes;c__Bacteroidia;o__Bacteroidales;f__Rikenellaceae;g__
k__Bacteria;p__Firmicutes;c__Clostridia;o__Clostridiales;f__Lachnospiraceae;g__[Ruminococcus]
k__Bacteria;p__Proteobacteria;c__Gammaproteobacteria;o__Enterobacteriales;f__Enterobacteriaceae;g__
k__Bacteria;p__Proteobacteria;c__Betaproteobacteria;o__Burkholderiales;f__Alcaligenaceae;g__Sutterella
k__Bacteria;p__Bacteroidetes;c__Bacteroidia;o__Bacteroidales;f__Prevotellaceae;g__Prevotella
k__Bacteria;p__Firmicutes;c__Clostridia;o__Clostridiales;f__Ruminococcaceae;g__Ruminococcus
k__Bacteria;p__Firmicutes;c__Clostridia;o__Clostridiales;f__Lachnospiraceae;g__Blautia
k__Bacteria;p__Firmicutes;c__Bacilli;o__Lactobacillales;f__Lactobacillaceae;g__Lactobacillus
Unassigned;Other;Other;Other;Other;Other
k__Bacteria;p__Firmicutes;c__Clostridia;o__Clostridiales;f__Lachnospiraceae;g__Dorea
k__Bacteria;p__Firmicutes;c__Clostridia;o__Clostridiales;f__Lachnospiraceae;g__Coprococcus
k__Bacteria;p__Firmicutes;c__Clostridia;o__Clostridiales;f__Dehalobacteriaceae;g__Dehalobacterium
k__Bacteria;p__Actinobacteria;c__Actinobacteria;o__Actinomycetales;f__Actinomycetaceae;g__Actinomyces
k__Bacteria;p__Firmicutes;c__Clostridia;o__Clostridiales;Other;Other
k__Bacteria;p__Firmicutes;c__Clostridia;o__Clostridiales;f__Peptococcaceae;g__rc4-4
k__Bacteria;p__Firmicutes;c__Bacilli;o__Turicibacterales;f__Turicibacteraceae;g__Turicibacter
k__Bacteria;p__Actinobacteria;c__Coriobacteriia;o__Coriobacteriales;f__Coriobacteriaceae;g__Adlercreutzia
k__Bacteria;p__Firmicutes;c__Clostridia;o__Clostridiales;f__Ruminococcaceae;Other
k__Bacteria;p__Firmicutes;c__Clostridia;o__Clostridiales;f__Peptostreptococcaceae;g__
